# Modeling the Impact of Climatological Factors and Technological Revolution on Soybean Yield: Evidence from 13-Major Provinces of China

**DOI:** 10.3390/ijerph19095708

**Published:** 2022-05-07

**Authors:** Huaquan Zhang, Abbas Ali Chandio, Fan Yang, Yashuang Tang, Martinson Ankrah Twumasi, Ghulam Raza Sargani

**Affiliations:** College of Economics, Sichuan Agricultural University, Chengdu 611130, China; zhanghuaquan@sicau.edu.cn (H.Z.); yangfan1@stu.sicau.edu.cn (F.Y.); 2020208002@stu.sicau.edu.cn (Y.T.); twuma2012@sicau.edu.cn (M.A.T.); razasargani@sicau.edu.cn (G.R.S.)

**Keywords:** climate change, technological progress, soybean yield, China

## Abstract

In recent years, the changing climate has become a major global concern, and it poses a higher threat to the agricultural sector around the world. Consequently, this study examines the impact of changing climate and technological progress on soybean yield in the 13 major provinces of China, and considers the role of agricultural credit, farming size, public investment, and power of agricultural machinery from 2000 to 2020. Fully modified ordinary least squares (FMOLS) and dynamic ordinary least squares (DOLS) are applied to assess the long-run effect, while Dumitrescu and Hurlin’s (2012) causality test is used to explore the short-run causalities among the studied variables. The results revealed that an increase in the annual mean temperature negatively and significantly affects soybean yield, while precipitation expressively helps augment soybean yield. Furthermore, technological factors such as chemical fertilizers accelerate soybean yield significantly, whereas pesticides negatively influence soybean yield. In addition, farming size, public investment, and power of agricultural machinery contribute remarkably to soybean yield. The causality results endorse that chemical fertilizers, pesticides used, agricultural credit, public investment, and power of agricultural machinery have bidirectional causality links with soybean yield. This study suggests several fruitful policy implications for sustainable soybean production in China.

## 1. Introduction

The phenomenon of climatic variations and their related effects, adaptation, and vulnerability have become a primary societal concern [1]. The literature has not quantified the extent of climate change safety, but it is clear that climate change will bring unexpected disasters to human beings and ecosystems. According to the report of C3S (Copernicus Climate Change Service) which aims to inform, raise awareness and provide data about climate change, 2021 was the warmest year in terms of global temperatures, and the global average temperature is 0.3 °C higher than what it was from 1991 to 2020.

Moreover, the Intergovernmental Panel on Climate Change (IPCC) reported that the 1.5 °C limit set by global leaders in 2015 is likely to be breached by 2030. This means that a warm global climate in the future is very likely to occur [2]. Thus, it has become ever more urgent to analyze the impacts of global warming. The rise in the concentration of greenhouse gases due to the continuing growth of the population is considered the primary cause of this warming. Additionally, other factors such as human activities, fossil fuel consumption, and deforestation have caused massive emissions [3]. Furthermore, researchers have found that although the role of fertilizers in agricultural production is profound, their excessive usage can lead to severe issues, including greenhouse gases [4]. For example, the accumulation of heavy metals and radionuclides is caused by excessive use of organic fertilizers, which directly lead to water, soil, and air pollution [5]. As a result, global warming has had a detrimental effect on developed and developing economies. Additionally, the agriculture sector is mainly affected when it comes to climate change venerability. Many studies have shown that changes in climatic conditions (e.g., rainfall, high temperatures, floods, droughts, and storms) significantly affect agricultural productivity [6,7,8]. Thus, crop yield is likely to decline due to changes in climate. Therefore, to ensure sustainable food production and food security, there is a need to promote more efficient and sustainable climatic coping and adaptation strategies.

A large number of studies have found that the growth in crop production is dependent upon the expansion and promotion of agricultural technology. Therefore, the popularization of agriculture mechanization and its related technological advancement activities (e.g., proper fertilizer use) are vital in ensuring efficiency in agricultural production [9]. Additionally, due to limited resources, continuous reduction in arable land, and increasing population growth, fertilizer has become a vital driving force in boosting crop yields since it enhances soil properties and increases soil fertility [10]. However, as we mentioned above, the inefficient use of fertilizers can lead to a higher cost. As a result, research has suggested that fertilizer should be applied efficiently and effectively to reduce environmental chaos while improving yields [11]. Furthermore, the advancement of agricultural technology plays an essential and positive role in the production of crops, for instance, plowing, planting, and harvesting [12,13]. Modern agricultural mechanization could contribute to the rapid growth of agricultural production, promote the application of energy-saving and reduce carbon emissions [14]. Moreover, some researchers stated that innovative agricultural technologies could enhance agriculture sustainability [15]. In general, the popularization of these advanced agricultural technologies has a potential positive role in ensuring food production and food security. Additionally, the yields of major food crops increase when farmers’ adopt and efficiently use modern agricultural technologies to accomplish a higher production within limited resources. Several advanced agricultural technologies, including improved seeds, chemical fertilizers, and water management technology, increase productivity and significantly improve farmers’ income.

Nowadays, dynamic changes in the climate harm the yield of significant food and oil crops and pose a threat to food security, particularly in developing countries. As one of the fastest developing countries, China is the largest grain producer globally, and its complex topography is highly vulnerable to climate change. In 2020, China’s total grain output hit 669.5 billion kg; thus, it was raised by 0.9% compared to the previous year [16]. In contrast to the reality of limited soybean supply, milk production demand in China continues to grow compared to the global average [17]. China is considered a significant soybean consumer globally, indicating a substantial domestic soybean supply and demand gap in the nation [17,18,19]. Before 2004, China’s soybean import volume was below 20.2 Mt, but it significantly rose to 86.7 Mt in 2015, thus, recording a total consumption of 86.9% [20]. The importation of soybean from countries such as the United States, Brazil, and Argentina has become necessary to meet the domestic demand. Higher foreign dependence makes Chinese soybeans more vulnerable to external adverse factors and increases soybean production risk.

From the domestic perspective, soybean production is mainly concentrated in three provinces and one central region where favorable weather occurs, such as low temperature and little sunshine, rain, and snow [19]. However, from a worldwide perspective, weather conditions such as EI Nino will continue to affect global climate change, and the Chinese soybean will be affected adversely by climatic changes [19]. Recognizing these problems led the Chinese government to provide several policies to promote soybean production in 2020. For example, an initiative to encourage new agronomic technologies (e.g., planting of high-quality) is a significant policy intervention introduced by the central government [2].

**Figure 1:**
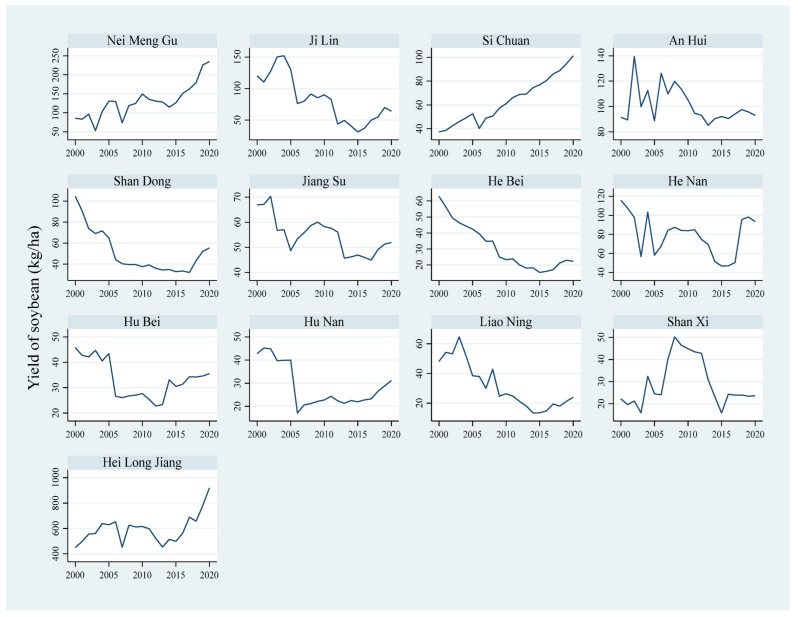
Major soybean-producing provinces of China, trends from 2000 to 2020.

In Figure 1, the upward trend in three soybean-producing provinces of China, namely Nei Meng Gu, Sichuan, and Hei Long Jiang can be seen, primarily due to favorable climate and adoption of new technologies, while in Ji Lin, An Hui, Shan Dong, Jiang Su, He Bei, Liao Ning, and Shan Xi soybean yield has been decreasing recently, due to climate variability and lack of resources (see Figure 1). Specifically, this figure suggests the trend of rapid decline in soybean yields in Shandong and relatively mild drops in Jiangsu, Hebei, and Liaoning. Its decline could be due to the impact of the international grain trade [21]. China is the world’s largest importer of soybeans. Hence, its large number of soybean imports has caused a decline in domestic soybean supply, which significantly impacts soybean yield in the above-mentioned coastal provinces. Anhui, Shanxi, and Henan are the main crop-producing areas in China, but their soybean production shows a decreasing trend. The cause of this phenomenon could be the low efficiency in soybean production compared with other crops [19], which has led to a decline in farmers’ interest in planting and a reduction in the yields. Together, the general provinces experienced encouraging outcomes in soybean planting; the soybean yields show provincial heterogeneny based on local specifics [22].

Several empirical studies related to the impacts of climatic change on agricultural products have been conducted in China in the last few years. For instance, Pickson et al. [23] and He et al. [24] assessed how climate variability influences cereal production. Additionally, while Pickson et al. [25] assessed how changing climate influences rice yield, Chandio et al. [26] and Rehman et al. [27] studied the influence of GHGs emissions on agricultural output. Again, the dynamic nexus between finance from the bank and climatic changes’ effects on agricultural production has been explored by Chandio et al. [28]. Wu et al. [11] also examined how mechanization and climatic changes affect grain productivity. From the literature, previous studies have not explored the impacts of climate variability and technological progress on soybean yield in the context of the 13 major provinces of China. To fill this gap, the present study attempts to test the long-run impact of climatic variables (i.e., temperature and precipitation) and technological factors (i.e., chemical fertilizers and pesticides used) on soybean cultivation in 13 major provinces (*Nei Meng Gu, Ji Lin, Sichuan, An Hui, Shan Dong, Jiang Su, He Bei, He Nan, Hu Bei, Hu Nan, Liao Ning, Shan Xi, and Hei Long Jiang*) of China. All 13 major provinces of China have a remarkable position for farming size and soybean yield in the context of China. Thus, this research uses panel data at the provincial level, not national data, and checks the dynamic linkages among the considered variables. The current research contributes to the emerging literature in the following ways: *Firstly*, this study uses the provincial data and inspects the effect of climate variability and technological progress on soybean yield. *Secondly*, this study estimates the effective role of farm size, agricultural credit, public investment, and agricultural power consumption. *Thirdly*, this paper applies a series of second-generation panel methods. *Finally*, this paper explores the causality among the yield of soybean, temperature, rainfall, fertilizers, pesticides, farm size, agricultural credit, public investment, and agricultural power consumption.

The remaining sections of this research include a review of the literature (Section 2), data and model construction (Section 3) estimations strategy and empirical results (Section 4), and a conclusion and policy implications (Section 5).

## 2. Literature Review

The dynamic changes in climate and their impact on the economy and human life have become a common concern for governments, society, and scientific circles. The Food and Agriculture Organization (FAO) stated that global climate change is a significant challenge for developed and developing economies. There is an urgent need to solve it and ensure food security. Many researchers have carried out plenty of theoretical and experimental studies to evaluate the effects of changing climate on soybean yield.

Most recent studies indicate that growth and development in crops, cropping systems and yield quality are all influenced by climate change in diverse ways. Parry et al. [29] showed that spatial effects play a significant role in climate change, affecting agricultural systems. Thus, in terms of regions, the research showed that temperate regions might experience climate change as a positive element of global warming, but the tropical regions’ experience may be negative. On the one side, some researchers such as Bhattarai et al. [30] and Tao et al. [31] concluded that agriculture might benefit from climate change, especially in the northeast of China, where the warmer temperature could extend the growing period of the crop and decrease the influence of frost damage on beans.

On the other side, in the existing literature, Challinor et al. [32] and Satari Yuzbashkandi and Khalilian [33] revealed that climate change might adversely affect soybean productivity and food security. These adverse effects of climate change are mainly associated with extremely high temperatures, such as evapotranspiration, availability of water, hikes in temperature of sowing and harvesting dates, and variation in precipitation patterns, all of which are harmful to crop growth. For example, using time-series data within the period of 1977 to 2014 and the autoregressive distributed lag-bounds (ARDL) bound testing method, a study in Pakistan showed that climate variations (e.g., temperature and rainfall patterns) were significant determining factors of cereal production [34].

The same story was told by Jan et al. [35]. After using average temperature and precipitation as a proxy for climatic changes and the second generation of panel cointegration analytical method for a time-series analysis from 1986 to 2015, they revealed that cereal production (wheat and maize) is positively influenced by increasing precipitation; however, in the long run, an insignificant impact of the rising average temperature was observed. The study recommended that the government increase the area under cultivation and improve irrigation systems and information accessibility for farmers to reduce the drastic impacts of climate change. By employing the ARDL procedure and using China’s quarterly data within the period of 1990Q1–2013Q4, Pickson et al. [23] examined cereal crop production and climate change linkage. The study showed that, in the long run, CO_2_, mean temperature, and temperature variability tremendously reduce production. However, energy, average precipitation, labor force, and farming size influenced the cereal crop production, which in the long run, was positive and significant.

He et al. [24] investigated the effects of global climate change (via temptarure and rainfall) on food crops production in Sichuan, China, covering the period from 1978 to 2018. The findings of the investigation revealed that temperature significantly and negatively affects food crops’ production, while rainfall enhances it. By employing data from 1970 to 2014 from wheat growers in Henan province, China, Zhai et al. [36] investigated the role of temperature and precipitation in wheat production. In conclusion, their study showed that rainfall decreases wheat production in the long term. Although there are two different voices, most scholars consider that the negative impacts outweigh the positive ones [37]. Therefore, to mitigate the dangerous impacts implications of climatic changes and improve the livelihoods of the vulnerable communities, Siamabele [38] suggests that the current world, and especially the emerging nations, needs practical adaptation actions.

Climate change has apparent direct effects on soybean yield, but technology is also one of the main influencing factors. Lybbert and Sumner [39] explored the expansion and effective diffusion of modern agricultural technologies and how they will largely shape the effects of climate change and adapt it. Additionally, Caetano et al. [40] provided evidence that new technologies, for instance, high-precision sowing, proper plant protection controls, and proper fertilizer formulas based on soil testing, can substantially improve agricultural production (e.g., soybean yields) and its sustainability. Furthermore, to understand the impact of technological advances, Tolassa et al. [41] also evaluated the relative effect of climate and agricultural machinery on actual soybean production in Brazilian municipalities. They found that technology advancement was positively associated with soybean productivity in Brazil.

As a proxy for technical progress, machinery and fertilizer use were seen to have a significant linkage to wheat productivity in China in the long run [36]. Based on field experiments in China, Henan Province, Wang et al. [42] investigated the relationship between technological advancement (irrigation and nitrogen fertilization usage) and wheat growth and yields. The study revealed that irrigation and nitrogen fertilization could improve wheat yield. Based on the existing research, the author argued that technological progression’s impact on agricultural production is profound; hence, analyzing the linkage between these two components is essential for policymakers.

Based on the previous studies, hypotheses (H1, H2, H3, and H4) to evaluate the impact of technological progress and climatic changes on soybean yield in 13 selected provinces of China were formulated as follows:

**Hypothesis** **(H1).** *Climate change (via temperature) significantly negatively affects soybean yield*.

**Hypothesis** **(H2).** *Climate change (via precipitation) significantly contributes toward soybean yield*.

**Hypothesis** **(H3).** *technological progress (via fertilizers used) significantly enhances soybean yield*.

**Hypothesis** **(H4).** *technological progress (via pesticides used) significantly negatively/positively affects soybean yield*.

## 3. Data and Model Construction

### 3.1. Data, Variables, and Descriptive Statistics

**Figure 2:**
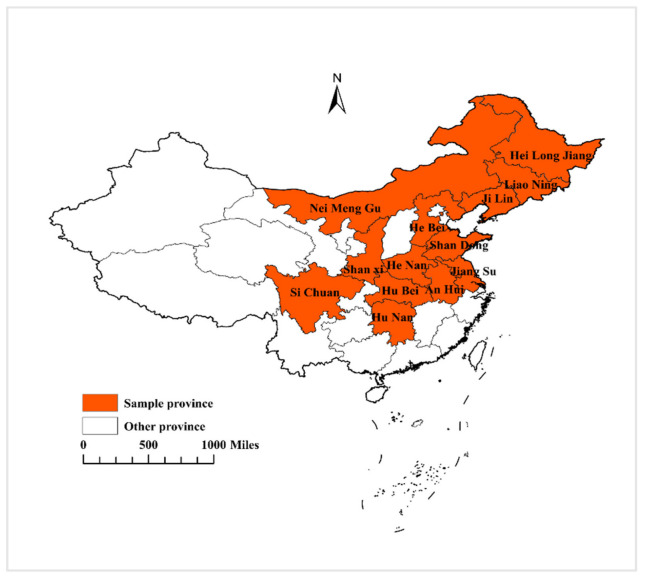
Map of the study area.

The present study consists of panel data of 13 selected soybean-producing provinces of China (see Figure 2), covering the period 2000–2020, which were acquired from the China Statistical Yearbook (CSY). In this investigation, we consider the soybean yield in kg/ha as the dependent variable, while the independent key factors are temperature, rainfall, fertilizer consumption, and pesticides used. Similarly to Abbas [43], Akhtar et al. [44], Chandio et al. [45], and Ozdemir [46], who used temperature and rainfall as a proxy of global climate change, we have also used the same proxies of climate change in our study. Similarly, Rehman et al. [47], Ali et al. [48], and Chandio et al. [49], who used fertilizer consumption and pesticides used to denote technological progress, we have considered the same technological factors in our investigation, whereas the control variables are farm size, agricultural credit, public investment, and agricultural power consumption. The measurements of studied variables and data sources are shown in Table 1.

The descriptive statistics of studied variables: soybean yield, temperature, rainfall, fertilizer consumption, pesticides, farm size, agricultural credit, public investment, and agricultural power consumption can be observed in Table 2. Preliminary results of Table 2 show that the mean value of the dependent variable lnsoyby is 4.0918. The average values of explanatory variables, including lntemp, lnrf, lnferc and lnfs, are 2.3899, 6.4813, 5.5275 and 8.7474. Further, the average values of other explanatory variables, such as lncredit, lnpinvet and lnagrpc are 7.6070, 5.3345 and 8.2550, respectively, and the standard deviations all of them show a symmetrical distribution. However, as shown in Table 2, the standard deviations of pestc reach up to 4.1510, representing a significant variation between the maximum and minimum.

### 3.2. Econometric Model

After gathering panel data from 13 provinces of China, we developed the main econometric function based on the previous studies [7,23,47]. Hence, the econometric model is expressed in Equation (1) to examine the association between soybean yield and its determinants, namely, temperature, rainfall, fertilizers consumption, pesticides, farm size, agricultural credit, public investment, and agricultural power consumption.

**Figure 3:**
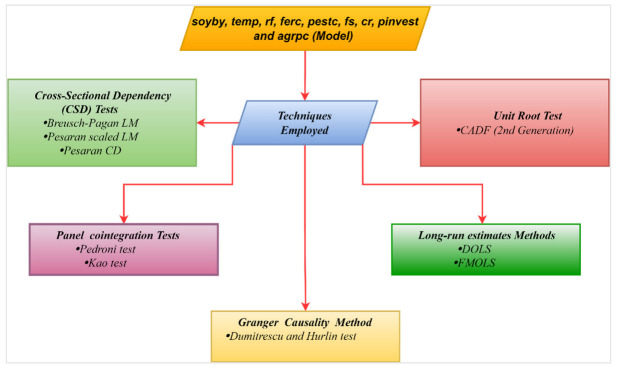
Steps of econometric strategy for the presenet study.

(1) soyby=f (temp, rf, ferc, pestc, fs, cr, pinvest, agrpc) 

where soyby is soybean yield, temp represents mean annual temperature, rf is mean yearly rainfall, ferc is the fertilizer consumption, pestc denotes the pesticides used, fs stands for farm size, cr refers the agricultural credit, pinvest indicates the public investment and agrpc shows the power consumption for agricultural machinery. Consequently, the underlying variables are transformed into natural logarithm forms, excluding pesticide usage, to acquire robust effects. After that, Equation (1) is reformulated into natural logarithm forms as follows:

(2) $$lnsoyby_{it\;}=\alpha_{0}+\beta_{1}lntemp_{it}+\beta_{2}lnrf_{it}+\beta_{3}lnferc_{it}+\beta_{4}pestc_{it}+\beta_{5}lnfs_{it}+\beta_{6}lncr_{it}+\beta_{7}lnpinvest_{it}+\beta_{8}lnagrpc_{it}+\varepsilon_{it}$$

where “i” and “t” represent the province-specific information and period of the panel dataset (2000 to 2020). In addition, “α” denotes the intercept while $\beta_{1},\beta_{2},\beta_{3},\beta_{4},\beta_{5},\beta_{6},\beta_{7},\;$ and $\;\beta_{8}$ indicate the coefficients of the long-run elasticity estimations of soybean yield with independent variables including temperature, precipitation, chemical fertilizers consumption, pesticides used, farm size cultivated under soybean, credit, public investment, and agricultural power consumption for agricultural machinery. In addition, Figure 3 demonstrates the econometric route of this investigation.

## 4. Estimations Strategy and Empirical Results

### 4.1. Cross-Sectional Dependency (CSD) and Panel Unit Root Test

The first step of this paper is to check the cross-sectional dependency (CSD) between the studied variables. Testing the CSD in the panel dataset before panel unit root analysis is supportive of applying appropriate techniques such as first generation or second generation procedures to tackle the issue of CSD in the panel dataset. Furthermore, by ignoring the CSD in the panel dataset estimation, all the estimated stationarity, cointegration, and regression results will be spurious [50,51]. Consequently, in the current study, we employed several cross-sectional dependence (CSD) tests, including Breusch–Pagan LM, Pesaran scaled LM, and Pesaran CD, to check the cross-sectional dependence among the considered variables that can be found generally in the economic data [52]. The results of CD tests proved that cross-sectional dependence exists between soybean yield, temperature, rainfall, fertilizers consumption, pesticide, farm size, agricultural credit, public investment, and agricultural power consumption, which rejects the null hypothesis at the 1% level of significance as presented in Table 3.

After the proof of CSD in the dataset, the panel unit root method is suggested to further test the stationary of the series. In this study, we applied the second generation panel unit root test, namely CADF, which can deal with the issue of CSD and eliminate the homogeneity issue [51]. Table 4 reports the current results of the CADF and indicates that lnferc and lnagrpc are stationary at their level, while lnsoyby, lntemp, lnrf, pesticide, lnfs, lncredit, and lnpinvet variables are stationary at their first difference.

### 4.2. Panel *Cointegration Analysis*

After checking the studied variables’ stationarity, the panel data investigation is needed to check the long-term linkage between the considered variables. We used Pedroni and Kao panel co-integration methods; results are reported in Table 5. The Pedroni and Kao cointegration techniques are used to test the null hypothesis (i.e., H0: no long-term cointegration exist) in contrast to the alternative hypothesis (i.e., H1: yes long-term cointegration exist). The current results of both tests have revealed strong evidence of the long-term cointegration among the underlying variables.

### 4.3. Long-Run Estimates and *Causality Analysis*

**Figure 4:**
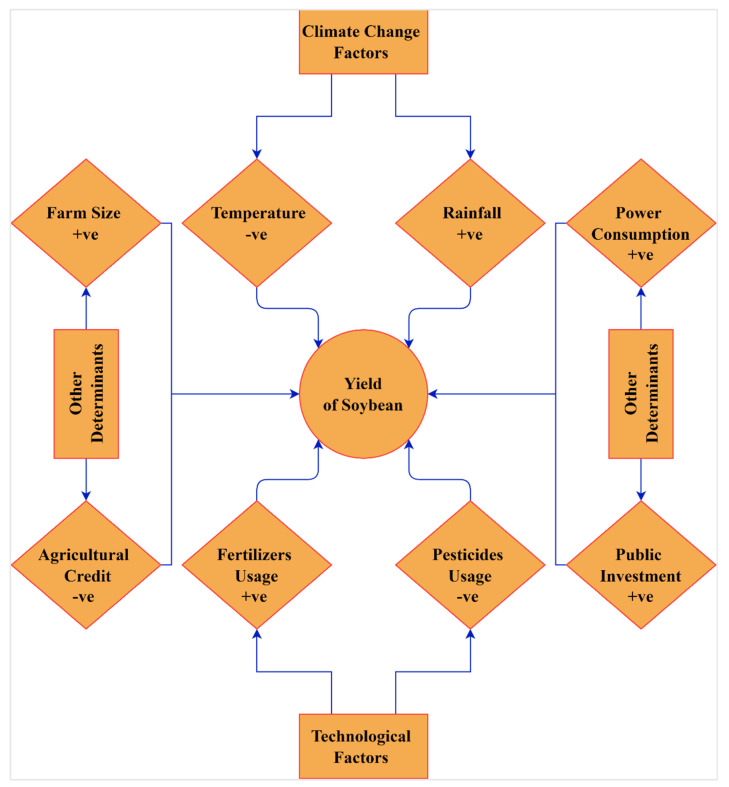
Summary of long-run estimates. “+ve” and “-ve” denote the positive effect and the negative effect, respectivley.

After establishing the co-integration relationship among the underlying variables, the next step of this investigation is to examine the long-run impact of temperature, rainfall, fertilizers consumption, pesticide, farm size, agricultural credit, public investment, and agricultural power consumption on soybean yield. The study employed the DOLS and FMOLS techniques since they are consistent long-run predictors. The results of both methods are presented in Table 6. The summary of long-run estimates is exhibited in Figure 4. The long-run linkage of diverse variables in soybean yield has depicted the same effects as in the DOLS and FMOLS; in other words, variables, whether in the DOLS or FMOLS has the same positive and negative impact on soybean yield.

Results from DOLS indicate that the increasing temperature has a highly significant and negative influence on soybean yield; this implies that an increase of 1% in temperature would reduce soybean yield by 0.979%, thus confirming *Hypothesis (H1)* of our study. Our results are consistent with Chen et al. [53], they used fine-scale meteorological data to find that increases in temperature above the thresholds will hurt crop yields; specifically, yields could increase with temperature up to 28 °C for soybean, but temperatures above these thresholds are very harmful to soybean growth. For example, in the northeast of China, increasing temperatures can prolong the growing period of the soybean and decrease the influence of frost damage on soybeans at the beginning. Furthermore, a Malaysian economic study influence of changing climate on palm oil crops, by Sarkar et al. [54], discovered that changing climate (via annual mean temperature) reduces oil palm productivity. At the same time, farming size enhances oil palm production after utilizing the OLS method and data from 1980 to 2010. Similar results were found by Abbas and Mayo [6], Pickson et al. [25], and Warsame et al. [55].

Furthermore, the long-run results reveal that both rainfall and fertilizer consumption have a significantly positive impact on soybean yield, increasing it by (0.721%) and (0.562%), respectively, consequently confirming both *Hypotheses* (i.e., *H2* and *H3)* of our study. Without a doubt, water is a critical resource for soybeans. On a regional scale and in a decadal period, obtained water comes primarily from rainfall. However, in northern China, climate change and its impacts on precipitation have caused drying, a cutoff in river flow, and shrinkage of lakes. The water shortage could directly lead to prolonged and repeated droughts and threaten food security in northern China [8,56,57]. Similarly, in the long run, fertilizers also play an enormously positive role in promoting the unit yields of soybean. At the early stages of plant growth, N-fixation may not supply enough N to meet plant demands. Most of the experimental results have shown that N-fertilizer would provide enough N for plant demand and increase the production of total dry matter, which can improve the potential of the plant to produce more pods, seeds, and ultimately, yields [56,58]. For instance, Abbas [43] examined the dynamic association between fertilizer, improved seed, cultivation area, and major cereals’ production in the case of Pakistan and revealed that usage of fertilizer and high-yield seeds are more beneficial for sustainable major cereals’ production.

The estimations of DOLS have presented that a 1% increase in pesticides brought a negative and significant change of 0.043% in soybean yield. In contrast, the estimates of FMOLS brought non-significant impacts, hence confirming *Hypothesis (H4)* of our study. The reason can be explained as follows: in agricultural production, the reasonable use of pesticides would promote farm output. In contrast, the excessive or unreasonable use of pesticides will partly reduce the security of soybean products, and harm environmental sustainability [57,58]. Next, the coefficient of farm size is beneficial to soybean production as it can be observed that a 1% change in the farm size will positively influence soybean production by 1.183%. In the persistence of small-scale farming size in China, many innovations from technology advancements, pathways of knowledge transfer to farmers, and new management skills are less effective due to high fixed costs of adoption [11,59]. Thus, Xu et al. [60] proposed that increasing the size of farms effectively improves per-worker soybean productivity in China. However, as shown in Table 6, a negative relationship between agricultural credit and soybean yields can be seen. When the supply of agricultural credit decreases by 1%, the soybean yields will reduce by 0.203% in the long term. It is generally acknowledged that rural households have no formal education, make no savings from their farming activities, and are unable to obtain credit information, so rural households are more likely to be refused credit when applied; refusing credit to rural households constrains their farming operations and makes them less productive [61]. For other explanatory variables, both public investment (0.085) and agricultural power consumption (0.060) positively influence soybean yield but are non-significant at the (*p* > 0.05) level.

Additionally, according to the FMOLS, estimates have shown similarly positive and negative impacts on soybean yields in the long run as those of DOLS, except for the coefficient size. Specifically, at a 1% upsurge in temperature, pesticides and agricultural credit will decrease soybean yields by 1.202%, 0.031%, and 0.356%, respectively. In contrast, when the rainfall, fertilizer consumption, farm size, public investment, and agricultural power consumption increase by 1%, the unit yields of soybean will increase by 1.055%, 0.817%, 0.958%, and 0.148%, and 0.301%, respectively (see Table 6).

**Figure 5:**
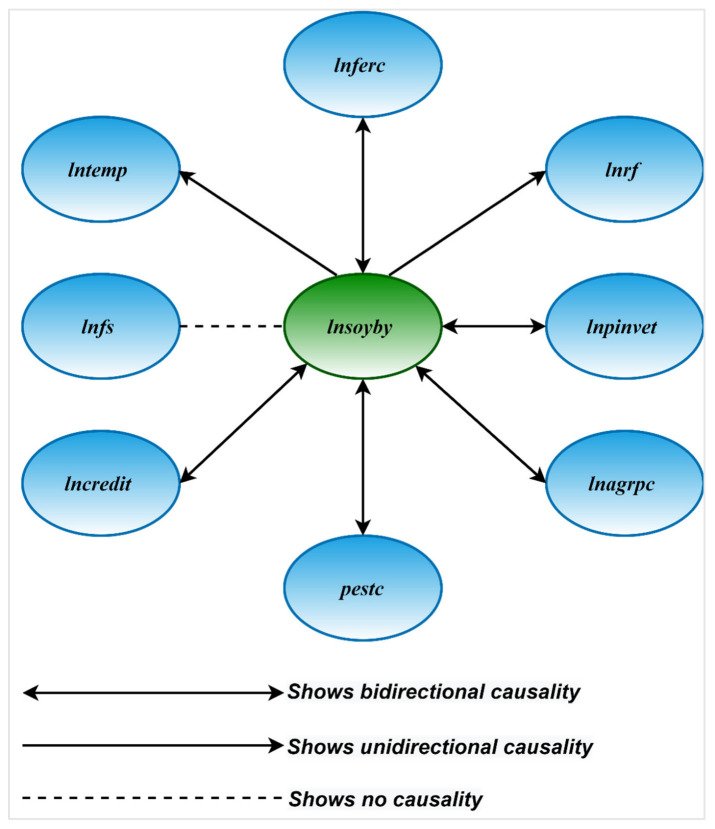
Key findings of the *D–H test.*

Finally, Table 7 reports the findings of the Dumitrescu and Hurlin panel causality investigation, which gauged the causal relationship among soybean yield, temperature, rainfall, fertilizer consumption, pesticides, farm size, agricultural credit, public investment, and agricultural power consumption. The findings show solid two-way causality of fertilizer consumption, pesticides, agricultural credit, public investment, and agricultural power consumption towards soybean yield. This means that technological factors (i.e., fertilizers consumption and pesticides) and other essential factors significantly influenced soybean yield in China’s significant soybean-producing provinces. On the other hand, our findings reveal a one-way causality of soybean yield towards climatic factors (i.e., temperature and rainfall). Figure 5 demonstrates the key outcomes of the D–H causality analysis.

## 5. Conclusions and Policy Implications

Soybean (Glycine max (L.) Merr.) is a staple food for Chinese people, and it is widely grown in several regions of China. Hence, the current paper assesses the impact of climate change (via temperature and rainfall) and technological factors (i.e., chemical fertilizers and pesticides used) on soybean yield in the context of central soybean-producing provinces of China. This study also incorporated other determinants of soybean yield, including farm size, agricultural credit, public investment, and power consumption for agricultural machinery. By applying an advanced series of practical tools that include CSD tests, CADF unit toot test, co-integration techniques, dynamic ordinary least squares (D-OLS), fully modified ordinary least squares (FM-OLS), and Dumitrescu and Hurlin causality test, interesting results revealed that: (1) Stationarity of the data and existence of the long-term co-integration among the variables have been proved. (2) Long-term FM-OLS and D-OLS show that temperature decreased the yield of soybean significantly. At the same time, precipitation expressively helps to increase the yield of soybean in central soybean-producing provinces of China. (3) Other determinants such as farm size, public investment, and power consumption for agricultural machinery positively contributed to soybean yield. This means that increasing farming size, public investment, and power of agricultural machinery will accelerate soybean yield and ensure the country’s food security. (4) The D-H causality method results show that fertilizer consumption, pesticides, agricultural credit, public investment, and agricultural power consumption have two-way causal connections with the yield of soybean. This means all factors significantly influenced the yield of soybean in the context of major soybean-producing provinces of China.

Based on the above results, some timely policies can be given to raise the soybean yield in China. It is clear that the policy implications of this article are affluent. First, climate change should be strictly tackled in China to protect soybean growth. To be specific, soybean yield is crucial in determining the economic and human development of the largest developing country, China. Hence, the Chinese government should cultivate beneficial environments for agricultural growth, including for the soybean. Mitigating climate change is an essential to providing favorable temperature and rainfall for soybean growth. Second, as far as is known, it is a challenge to deal with the climate change issue due to the vast geography of China. Further policies should be recommended based on local specifics instead of implementing one target for the whole country to reduce carbon emissions to a certain degree. Accordingly, this article provides a detailed snapshot by investigating the cross-provincial data to examine how climate change can influence soybean yield. The cross-regional database is essential to go one step further in understanding the heterogeneous effects of the associations between climate change and agricultural yields. In this case, local governments should implement specific policy designs according to their local climate change situations and soybean crops.

Third, technological revolutions are always significant in enhancing soybean yield. Therefore, multi-dimensional technological developments should be urgently applied in the agricultural industry in China. Specifically, advanced machines and chemical fertilizers should be further researched and applied in soybean cultivation. As explained by the results of this article, governments could have stronger motivations to invest in this sort of technological revolution in the near future. Fourth, the scale of economics should be considered for policy designs. As seen from our results, a larger farm size positively affects soybean yield, which suggests broadening the scale for growing soybeans is beneficial at the current stage for China. Still, this strategy should be mindful of being overly applied. Notably, timing and locations should be carefully considered before making political decisions regarding encouraging these plants. Further, financial support is important in increasing the yield of soybean. In this context, governments should encourage both public and private funds to enter the soybean markets of China, which could lead to a higher yield of soybean. Similarly, related laws and regulations could also assist the farmers in obtaining financial credit from both formal and informal channels.

## Figures and Tables

**Table 1:** The measurements of the studied variables and data source.

Variables	Symbol	Measurement	Source
Yield of soybean	*soyby*	kg/ha	CSY
Mean annual temperature	*temp*	Degree Celsius	CSY
Mean annual rainfall	*rf*	Mm	CSY
Fertilizers consumption	*ferc*	10,000 tons	CSY
Pesticides used	*pestc*	Tons	CSY
Farm size	*fs*	1000 ha	CSY
Agricultural credit	*cr*	RMB 100 million	CSY
Public investment	*pinvest*	RMB 100 million	CSY
Agricultural power consumption	*agrpc*	10,000 kilowatts	CSY

**Note:** CSY denotes the China Statistical Yearbook, while RMB stands for Renminbi.

**Table 2:** The descriptive statistics of the studied variables.

**Variables**	Mean	Std. Dev.	Min	Max
lnsoyby	4.0918	0.8886	2.5885	6.8246
lntemp	2.3899	0.5218	0.8329	2.9231
lnrf	6.4813	0.4707	5.2183	7.8702
lnferc	5.5275	0.4541	4.3141	6.5738
pestc	7.7633	4.1510	0.8900	17.3500
lnfs	8.7474	0.3544	7.9842	9.7523
lncredit	7.6070	1.7138	4.4434	10.5751
lnpinvet	5.3345	1.3065	2.2310	8.7098
lnagrpc	8.2550	0.6452	6.9230	9.4994

**Table 3:** Cross-sectional dependence testing results.

**Variables**	Breusch-Pagan LM	Pesaran Scaled LM	Pesaran CD
lnsoyby	311.6154 (0.0000)	17.6633 (0.0000)	5.2415 (0.0000)
lntemp	230.7756 (0.0000)	11.1910 (0.0000)	6.4063 (0.0000)
lnrf	173.0196 (0.0000)	6.5668 (0.0000)	6.6930 (0.0000)
lnferc	420.3057 (0.0000)	26.3655 (0.0000)	4.2402 (0.0000)
pestc	228.0176 (0.0000)	10.9701 (0.0000)	10.1171 (0.0000)
lnfs	359.0804 (0.0000)	21.4636 (0.0000)	5.9248 (0.0000)
lncredit	394.5919 (0.0000)	24.3068 (0.0000)	15.1850 (0.0000)
lnpinvet	455.5671 (0.0000)	29.1887 (0.0000)	15.0127 (0.0000)
lnagrpc	422.8512 (0.0000)	26.5693 (0.0000)	7.1716 (0.0000)

**Table 4:** Unit root testing results.

	CADF Test			
	Level	*p*-Value	Fist-Difference	*p*-Value
lnsoyby	−1.517	0.827	−3.039 ***	0.000
lntemp	−1.194	0.984	−3.463 ***	0.000
lnrf	−1.780	0.485	−2.302 **	0.026
lnferc	−2.278 **	0.029	−2.161 *	0.073
pestc	−2.010	0.186	−2.951 ***	0.000
lnfs	1.608	1.000	−3.519 ***	0.000
lncredit	−1.967	0.232	−3.006 ***	0.000
lnpinvet	−1.131	0.991	−2.850 ***	0.000
lnagrpc	−2.334 **	0.018	−2.345 **	0.015

**Note:** *** *p* < 0.01, ** *p* < 0.05, and * *p* < 0.1.

**Table 5:** Panel co-integration testing method.

**Pedroni Test**	**Panel Tests**	**Statistics**	*p*-Value
Within dimension	Panel PP-Stat	−3.055407 ***	0.0011
	Panel ADF-Stat	−3.079233 ***	0.0010
Between dimension	Group PP-Stat	−2.775922 ***	0.0028
	Group ADF-Stat	−2.859674 ***	0.0021
Kao Test	ADF t-Statistic	−2.275874 **	0.0114

**Note:** *** *p* < 0.01 and ** *p* < 0.05.

**Table 6:** Benchmark results.

**Variables**	Coef.	Std. Err.	z	*p* > z
DOLS				
lntemp	−0.979 ***	0.164	−5.960	0.000
lnrf	0.721 ***	0.103	7.010	0.000
lnferc	0.562 ***	0.171	3.280	0.001
pestc	−0.043 ***	0.014	−3.030	0.002
lnfs	1.183 ***	0.190	6.220	0.000
lncredit	−0.203 ***	0.056	−3.590	0.000
lnpinvet	0.085	0.068	1.260	0.208
lnagrpc	0.060	0.113	0.530	0.596
_Cons	−10.770 ***	1.666	−6.460	0.000
FMOLS				
lntemp	−1.202 **	0.478	−2.520	0.012
lnrf	1.055 ***	0.281	3.750	0.000
lnferc	0.817 *	0.473	1.730	0.084
pestc	−0.031	0.039	−0.800	0.425
lnfs	0.958 *	0.538	1.780	0.075
lncredit	−0.356 **	0.154	−2.310	0.021
lnpinvet	0.148	0.192	0.770	0.440
lnagrpc	0.301	0.318	0.950	0.344
_Cons	−12.394 ***	4.566	−2.710	0.007

**Note:** *** *p* < 0.01, ** *p* < 0.05, and * *p* < 0.1.

**Table 7:** D–H panel causality exploration.

Null Hypothesis:	W-Stat.	Zbar-Stat.	*p*-Value
lntemp does not homogeneously cause lnsoyby	1.27581	0.28890	0.7727
lnsoyby does not homogeneously cause lntemp	2.25402	2.27245	0.0231 **
lnrf does not homogeneously cause lnsoyby	0.69129	−0.89634	0.3701
lnsoyby does not homogeneously cause lnrf	4.06778	5.95028	3 × 10^−9^ ***
lnferc does not homogeneously cause lnsoyby	4.48484	6.79596	1 × 10^−11^ ***
lnsoyby does not homogeneously cause lnferc	6.72337	11.3351	0.0000 ***
pestc does not homogeneously cause lnsoyby	3.10588	3.99980	6 × 10^−5^ ***
lnsoyby does not homogeneously cause pestc	2.50988	2.79127	0.0053 ***
lnfs does not homogeneously cause lnsoyby	0.89766	−0.47788	0.6327
lnsoyby does not homogeneously cause lnfs	0.76569	−0.74549	0.4560
lncredit does not homogeneously cause lnsoyby	2.36589	2.49930	0.0124 **
lnsoyby does not homogeneously cause lncredit	2.51493	2.80151	0.0051 ***
lnpinvet does not homogeneously cause lnsoyby	3.01979	3.82523	0.0001 ***
lnsoyby does not homogeneously cause lnpinvet	3.84148	5.49141	4 × 10^−8^ ***
lnagrpc does not homogeneously cause lnsoyby	3.98780	5.78810	7 × 10^−9^ ***
lnsoyby does not homogeneously cause lnagrpc	3.19739	4.18535	3 × 10^−5^ ***

**Note:** *** *p* < 0.01 and ** *p* < 0.05.

## Data Availability

The data will be available on request.

## References

[1] Luo Q., Yu Q. (2012). Developing higher resolution climate change scenarios for agricultural risk assessment: Progress, challenges and prospects. Int. J. Biometeorol..

[2] Li R.-L., Geng S. (2013). Impacts of climate change on agriculture and adaptive strategies in China. J. Integr. Agric..

[3] Kang Y., Khan S., Ma X. (2009). Climate change impacts on crop yield, crop water productivity and food security–A review. Prog. Nat. Sci..

[4] Guo L., Li H., Cao X., Cao A., Huang M. (2021). Effect of agricultural subsidies on the use of chemical fertilizer. J. Environ. Manag..

[5] Lobell D.B., Burke M.B., Tebaldi C., Mastrandrea M.D., Falcon W.P., Naylor R.L. (2008). Prioritizing climate change adaptation needs for food security in 2030. Science.

[6] Abbas S., Mayo Z.A. (2021). Impact of temperature and rainfall on rice production in Punjab, Pakistan. Environ. Dev. Sustain..

[7] Bhardwaj M., Kumar P., Kumar S., Dagar V., Kumar A. (2022). A district-level analysis for measuring the effects of climate change on production of agricultural crops, ie, wheat and paddy: Evidence from India. Environ. Sci. Pollut. Res..

[8] Xie W., Huang J., Wang J., Cui Q., Robertson R., Chen K. (2020). Climate change impacts on China’s agriculture: The responses from market and trade. China Econ. Rev..

[9] Cassman K.G., Dobermann A., Walters D.T. (2002). Agroecosystems, nitrogen-use efficiency, and nitrogen management. AMBIO A J. Hum. Environ..

[10] Jiang Z., Zheng H., Xing B. (2021). Environmental life cycle assessment of wheat production using chemical fertilizer, manure compost, and biochar-amended manure compost strategies. Sci. Total Environ..

[11] Wu Z., Dang J., Pang Y., Xu W. (2021). Threshold effect or spatial spillover? The impact of agricultural mechanization on grain production. J. Appl. Econ..

[12] Belton B., Win M.T., Zhang X., Filipski M. (2021). The rapid rise of agricultural mechanization in Myanmar. Food Policy.

[13] Chuanqi H., Jing J. (2017). Challenges and Policy suggestions for China’s Agricultural Modernization. Mod. Sci. Newsl..

[14] He P., Zhang J., Li W. (2021). The role of agricultural green production technologies in improving low-carbon efficiency in China: Necessary but not effective. J. Environ. Manag..

[15] Ennouri K., Kallel A. (2019). Remote sensing: An advanced technique for crop condition assessment. Math. Probl. Eng..

[16] Li W., Zhang P. (2021). Relationship and integrated development of low-carbon economy, food safety, and agricultural mechanization. Environ. Sci. Pollut. Res..

[17] Bai Z., Ma W., Ma L., Velthof G.L., Wei Z., Havlík P., Oenema O., Lee M.R., Zhang F. (2018). China’s livestock transition: Driving forces, impacts, and consequences. Sci. Adv..

[18] Sheng Y., Song L. (2019). Agricultural production and food consumption in China: A long-term projection. China Econ. Rev..

[19] Zhang Z., Lu C. (2020). Clustering analysis of soybean production to understand its spatiotemporal dynamics in the North China Plain. Sustainability.

[20] Torres S.M., Moran E.F., da Silva R.F.B. (2017). Property rights and the soybean revolution: Shaping how China and Brazil are telecoupled. Sustainability.

[21] Ren D., Yang H., Zhou L., Yang Y., Liu W., Hao X., Pan P. (2021). The Land-Water-Food-Environment nexus in the context of China’s soybean import. Adv. Water Resour..

[22] Zhao J., Wang C., Shi X., Bo X., Li S., Shang M., Chen F., Chu Q. (2021). Modeling climatically suitable areas for soybean and their shifts across China. Agric. Syst..

[23] Pickson R.B., He G., Ntiamoah E.B., Li C. (2020). Cereal production in the presence of climate change in China. Environ. Sci. Pollut. Res..

[24] He W., Chen W., Chandio A.A., Zhang B., Jiang Y. (2022). Does Agricultural Credit Mitigate the Effect of Climate Change on Cereal Production? Evidence from Sichuan Province, China. Atmosphere.

[25] Pickson R.B., He G., Boateng E. (2021). Impacts of climate change on rice production: Evidence from 30 Chinese provinces. Environ. Dev. Sustain..

[26] Chandio A.A., Jiang Y., Rehman A., Rauf A. (2020). Short and long-run impacts of climate change on agriculture: An empirical evidence from China. Int. J. Clim. Chang. Strateg. Manag..

[27] Rehman A., Ma H., Irfan M., Ahmad M. (2020). Does carbon dioxide, methane, nitrous oxide, and GHG emissions influence the agriculture? Evidence from China. Environ. Sci. Pollut. Res..

[28] Chandio A.A., Jiang Y., Abbas Q., Amin A., Mohsin M. (2020). Does financial development enhance agricultural production in the long-run? Evidence from China. J. Public Aff..

[29] Parry M.L., Canziani O., Palutikof J., Van der Linden P., Hanson C. (2007). Climate Change 2007-Impacts, Adaptation and Vulnerability: Working Group II Contribution to the Fourth Assessment Report of the IPCC.

[30] Bhattarai M.D., Secchi S., Schoof J. (2017). Projecting corn and soybeans yields under climate change in a Corn Belt watershed. Agric. Syst..

[31] Tao F., Yokozawa M., Liu J., Zhang Z. (2008). Climate–crop yield relationships at provincial scales in China and the impacts of recent climate trends. Clim. Res..

[32] Challinor A.J., Ewert F., Arnold S., Simelton E., Fraser E. (2009). Crops and climate change: Progress, trends, and challenges in simulating impacts and informing adaptation. J. Exp. Bot..

[33] Yuzbashkandi S.S., Khalilian S. (2020). On projecting climate change impacts on soybean yield in Iran: An econometric approach. Environ. Processes.

[34] Chandio A.A., Magsi H., Ozturk I. (2020). Examining the effects of climate change on rice production: Case study of Pakistan. Environ. Sci. Pollut. Res..

[35] Jan I., Ashfaq M., Chandio A.A. (2021). Impacts of climate change on yield of cereal crops in northern climatic region of Pakistan. Environ. Sci. Pollut. Res..

[36] Zhai S., Song G., Qin Y., Ye X., Lee J. (2017). Modeling the impacts of climate change and technical progress on the wheat yield in inland China: An autoregressive distributed lag approach. PLoS ONE.

[37] Li S., You S., Song Z., Zhang L., Liu Y. (2021). Impacts of Climate and Environmental Change on Bean Cultivation in China. Atmosphere.

[38] Siamabele B. (2021). The significance of soybean production in the face of changing climates in Africa. Cogent Food Agric..

[39] Lybbert T.J., Sumner D.A. (2012). Agricultural technologies for climate change in developing countries: Policy options for innovation and technology diffusion. Food Policy.

[40] Caetano J.M., Tessarolo G., De Oliveira G., Souza K.D.S.E., Diniz-Filho J.A.F., Nabout J.C. (2018). Geographical patterns in climate and agricultural technology drive soybean productivity in Brazil. PLoS ONE.

[41] Tolasa M., Gedamu F., Woldetsadik K. (2021). Impacts of harvesting stages and pre-storage treatments on shelf life and quality of tomato (*Solanum lycopersicum* L.). Cogent Food Agric..

[42] Wang J.-X., Huang J.-K., Jun Y. (2014). Overview of impacts of climate change and adaptation in China’s agriculture. J. Integr. Agric..

[43] Abbas S. (2022). Climate change and major crop production: Evidence from Pakistan. Environ. Sci. Pollut. Res..

[44] Akhtar R., Masud M.M. (2022). Dynamic linkages between climatic variables and agriculture production in Malaysia: A generalized method of moments approach. Environ. Sci. Pollut. Res..

[45] Chandio A.A., Jiang Y., Amin A., Ahmad M., Akram W., Ahmad F. (2021). Climate change and food security of South Asia: Fresh evidence from a policy perspective using novel empirical analysis. J. Environ. Plan. Manag..

[46] Ozdemir D. (2021). The Impact of Climate Change on Agricultural Productivity in Asian Countries: A heterogeneous panel data approach. Environ. Sci. Pollut. Res..

[47] Rehman A., Chandio A.A., Hussain I., Jingdong L. (2019). Fertilizer consumption, water availability and credit distribution: Major factors affecting agricultural productivity in Pakistan. J. Saudi Soc. Agric. Sci..

[48] Ali S., Liu Y., Nazir A., Ishaq M., Khan S., Abdullah S.T., Shah T. (2020). Does technical progress mitigate climate effect on crops yield in Pakistan. J. Anim. Plant Sci..

[49] Chandio A.A., Jiang Y., Akram W., Adeel S., Irfan M., Jan I. (2021). Addressing the effect of climate change in the framework of financial and technological development on cereal production in Pakistan. J. Clean. Prod..

[50] Xiaoman W., Majeed A., Vasbieva D.G., Yameogo C.E.W., Hussain N. (2021). Natural resources abundance, economic globalization, and carbon emissions: Advancing sustainable development agenda. Sustain. Dev..

[51] Usman M., Makhdum M.S.A. (2021). What abates ecological footprint in BRICS-T region? Exploring the influence of renewable energy, non-renewable energy, agriculture, forest area and financial development. Renew. Energy.

[52] Baloch M.A., Wang B. (2019). Analyzing the role of governance in CO2 emissions mitigation: The BRICS experience. Struct. Chang. Econ. Dyn..

[53] Chen S., Chen X., Xu J. (2013). Impacts of Climate Change on Corn and Soybean Yields in China. Proceedings of the 2013 Annual Meeting.

[54] Sarkar M.S.K., Begum R.A., Pereira J.J. (2020). Impacts of climate change on oil palm production in Malaysia. Environ. Sci. Pollut. Res..

[55] Warsame A.A., Sheik-Ali I.A., Ali A.O., Sarkodie S.A. (2021). Climate change and crop production nexus in Somalia: An empirical evidence from ARDL technique. Environ. Sci. Pollut. Res..

[56] Caliskan S., Ozkaya I., Caliskan M., Arslan M. (2008). The effects of nitrogen and iron fertilization on growth, yield and fertilizer use efficiency of soybean in a Mediterranean-type soil. Field Crops Res..

[57] Ramteke R., Gupta G.K., Singh D.V. (2015). Growth and yield responses of soybean to climate change. Agric. Res..

[58] Janagard M.S., Raei Y., Gasemi-Golezani K., Aliasgarzad N. (2013). Soybean response to biological and chemical fertilizers. Int. J. Agric. Crop Sci..

[59] Wu Y., Xi X., Tang X., Luo D., Gu B., Lam S.K., Vitousek P.M., Chen D. (2018). Policy distortions, farm size, and the overuse of agricultural chemicals in China. Proc. Natl. Acad. Sci. USA.

[60] Xu Y., Xin L., Li X., Tan M., Wang Y. (2019). Exploring a moderate operation scale in China’s grain production: A perspective on the costs of machinery services. Sustainability.

[61] Ansah I.K., Toatoba J., Donkoh S. (2016). The effect of credit constraints on crop yield: Evidence from soybean farmers in Northern Region of Ghana. Ghana J. Sci. Technol. Dev..

